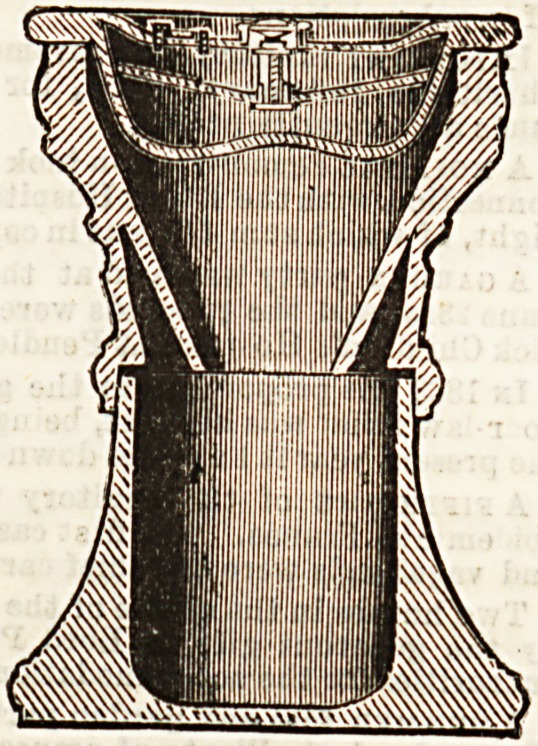# Chamber Portable Closet

**Published:** 1893-07-15

**Authors:** 


### CHAMBER PORTABLE CLOSET.

The inventor and patentee of this portable closet, of which we give two illustrations, is Mr. J. C. Daniel, 18, Pall Mall, Hanley, who has supplied an article which will be found extremely useful in the sick room. It is made of well-glazed earthenware, and thus, being non-absorbent, it is easy to be kept clean and pure, and is therefore especially to be recommended in cases of infectious illness. The section shows that the closet consists of three parts—a lower receiver, with an upper vessel, funnel-shaped, fitting closely into the first. The cover contains "a supply chamber for disinfecting fluid, and a distributing chamber, having its bottom perforated and shaped so as to evenly distribute any liquid passed into it, and connected with the supply chamber by a spring valve, which can be operated on by means of a knob. The supply chamber or cistern is filled with disinfecting fluid through the orifice, closed by a screw plug on the top of the cover." The closetiere cover can be procured and used separately if desired. The principal upon which Mr. Daniel has based his invention is good, and appears to be carried out in a simple and convenient manner. Nurses will welcome the portable closet, as it will undoubtedly be a great aid to thorough disinfection. The price is moderate.

## Figures and Tables

**Figure f1:**
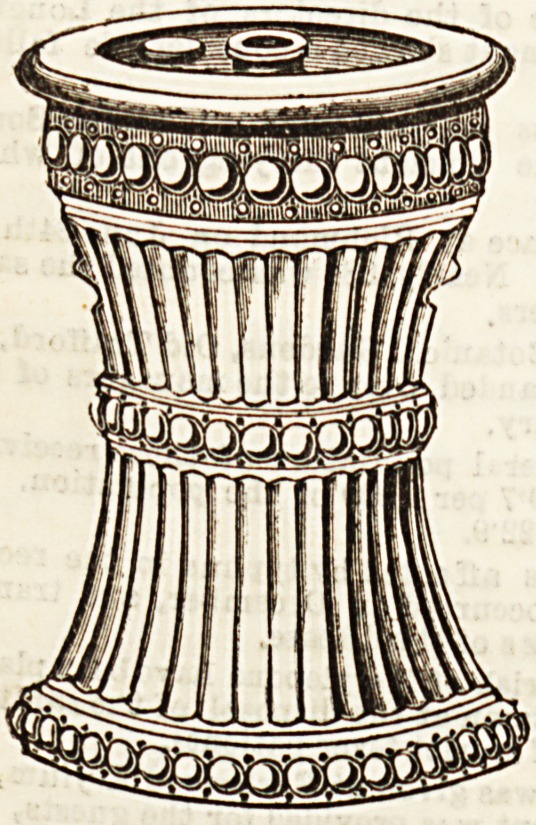


**Figure f2:**